# Comparison of Behavioral Concerns Priorities among Iranian Adolescent Boys, Parents and Teachers: Reporting the Results of a Modified Delphi

**DOI:** 10.30476/ijcbnm.2020.85222.1257

**Published:** 2021-01

**Authors:** Fatemeh Mohammadipour, Marjan Fathi, Manzar Amirkhani, Sarah Nouriyengejeh, Ata Pourabbasi

**Affiliations:** 1 Social Determinants of Health Research Center, Lorestan University of Medical Sciences, Khorramabad, Iran; 2 Islamic Azad University, Kish Branch, Iran; 3 Department of Counseling, Faculty of Educational Sciences, Tonekabon Branch, Islamic Azad University, Mazandaran, Iran; 4 Endocrinology and Metabolism Research Center, Endocrinology and Metabolism Clinical Sciences Institute, Tehran University of Medical Sciences, Tehran, Iran

**Keywords:** Adolescent, Behavioral concerns, Delphi, Iran, Parent, Prioritization

## Abstract

**Background:**

Adolescence is a time of risks and opportunities. This study aimed to investigate and prioritize the behavioral concerns of male adolescents.

**Methods:**

A modified Delphi study (2018-2019) was conducted in two stages. In the first stage, adolescents’ behavioral concerns were extracted based on seven qualitative interviews and a focus group. Then, a questionnaire was designed using the obtained data; also, two-round consensus-building approach (rating and ranking) through surveys were conducted among 90students, teachers and parents covered by the Health Departments of Tehran University of Medical Sciences, who had been selected using a stratified cluster random sampling method. Qualitative content analysis was used in the first stage and descriptive statistics in the second stage to analyze the data.

**Results:**

In the first stage, eight categories emerged, including relational challenges with parents and other adults; poor interaction with peers; lack of adaptation to conditions; emotional dysregulation; self-esteem and sense of purposefulness issues; materialistic tendencies; cyberspace issues; and non-adherence to religious beliefs. Then 63 behavioral concerns were identified. In each round (rating/ranking), 29/27, 28/29, and 30/30, responses were received for students, parents and teachers, respectively. According to the specified scores given to different issues, ten top problems according to the viewpoint of different groups were extracted.

**Conclusion:**

“lack of planning and prioritization skill”, “insufficient skill for controlling crisis”, and “lack of resilience” are the most important priority for male adolescents, teachers and parents, respectively. These findings can be used for planning programs based on the adolescents’ behavioral concerns.

## INTRODUCTION

Adolescence is a time of risks and opportunities, ^1^ a critical phase that sets the foundation of adulthood. ^2^ Some psychologists believe that the safe transition of adolescents from this phase will pave the way for a safe maturity phase and a prosperous social life. However, if the individual is confronted with some problems during this critical phase, his mental health will be affected inevitably. Such being the case, the adolescent will be misguided to a No End way. ^3^

At this phase, some adolescents may have negative tendency towards risky behaviors such as smoking, using drugs or alcohol, having unhealthy sexual relations, truancy, or other felonious behaviors which may be associated with negative effects on their mental and physical health. ^4^

Clearly, owing to the problems of adolescence, these groups of individuals are described as those at the frontier of hazards of behavioral problems and mental pressures. ^5^ According to statistics provided by the Statistical Center of Iran, there are 23 million adolescents in Iran. ^6^ The average of adolescents with behavioral problems is estimated to be 13.4%. Among these, about 10% need to receive mental health counseling. ^7^ A higher number of male adolescents are confronted with the behavioral disorders compared to their female counterparts. ^4^ In this respect, some studies have been conducted in different parts of Iran, which have estimated the prevalence of these behavioral disorders about 10-27.1%. The researchers of these studies have found that behavioral problems are more prevalent among boys. Some of these problems are those reported in relation to the quality of their communication with peers, parents, as well as struggles at school and society in addition to their private issues. ^3 , 8 , 9^ In addition, it is noteworthy that the prevalence of these problems and their nature can vary among different cultures and subcultures. These differences call for different intervention strategies for each group. ^1 , 10^

Despite the complex behavioral challenges to which adolescent boys are faced in contemporary society, there is remarkably limited information about their priority and particular educational needs. In order to understand this, it is essential that the viewpoint of male adolescents themselves be asked in relation to identifying their behavioral concerns and their sources of knowledge about these. ^11^ Although research in other countries could provide some helpful data, the extent of religious, socio-cultural and political differences, as well as different availability of appropriate resources reveals that each country needs its own research data in order to better understand how the behavioral concerns of young boys can be best met. This is congruent with the World Health Organization (WHO), stating that national level data is essential to inform national decision makers in relation to the most relevant policies to support young people.12 For example, a study conducted on the differences in the youth’s reported management of anger in US, Kenya, and Ghana found that youth from Kenya and the United States reported less overt anger expression than Ghanaian youth. ^13^ Similarly, a study about emotion regulation and psychopathology in Iranian and German school children showed that Iranian children report more internalizing and externalizing symptoms. It seems to be difficult for in Iranian young children express themselves. ^14^ While there has been much improvement in primary health care in Iran, the particular behavioral concerns of adolescents have been neglected. ^11^ This lack of limited information necessitates a rigorous study to examine and prioritize behavioral concerns in high schools.

Delphi method is an alternative way through which to gather expert opinion on what challenges are likely to be most priority in this context, and is often used when evidence or scientific knowledge is lacking. ^15^

Because of the importance of this phase, ^4^ the point that the behaviors in next phase of life are established during the adolescence, ^16^ and considering the fact that such problems are reported to be more prevalent among boys, ^12^ the modified Delphi study was designed to determine the behavioral priorities among Iranian adolescent boys, parents and teachers. 

## MATERIALS AND METHODS

Delphi methodology, by using a multistage self-completed questionnaire with individual feedback, allows expert opinion to be obtained when little is known about a topic. ^17 , 18^ The original classic Delphi consists of four rounds: generation of ideas, construction of the survey instrument, evaluation phase, and re-evaluation of original responses. However, these rounds can be modified by researchers to suit specific aims of the study and in some cases it has been shortened to two or three rounds by using focused groups or interviews as a substitute for the first qualitative round. ^19^ The modified Delphi is similar to the full Delphi in terms of procedure, but it consists a set of carefully pre-selected items drawn from various sources including interviews with experts. ^15 , 19^

In this study, a modified Delphi was employed in two stages. During the first stage, qualitative data were gathered from the participants. Throughout the subsequent rounds, data gathered from the first stage was presented to the panelist as an online questionnaire to obtain their feedback, agreement or disagreement. This study was conducted from July 2018 to November 2019 among the students, teachers and parents covered by the Health Departments of Tehran University of Medical Sciences in Tehran, Iran.

### First Stage: Primary Identification of Problems

A descriptive qualitative phase was designed for the primary identification of the adolescents’ behavioral concerns. This qualitative phase was necessary because there are no written documents which mention these problems and, as indicated in the introduction, there was a need to get the views of adolescents and their parents; on the other hand, school counselors are well aware of the students’ problems and challenges. Qualitative method is useful for explaining the issues and problems that need to be addressed. ^20^

The participants of this phase were selected through purposive sampling with maximum variation. The participants had a variety of education level, age and work experiences. First, deep semi-structured individual interviews with seven expert school counselors were conducted, each lasting 40-75 minutes. Data saturation was achieved after completing five individual interviews, but to ensure that no new themes were being missed, two other interviews were conducted. Then, a focused group consisting of ten adolescents and seven parents was formed. Interviews were conducted using an interview guide prepared based on the research goals. Some of interview questions were “what does behavioral concern of adolescents mean”; “can you explain the causes of behavioral problems of adolescents?” Then, branching questions were asked to improve the depth of the interviews. 

Data were analyzed using conventional content analysis which included transcribing the whole interviews, determining he analysis and meaning unit, simplifying the meaning unit, determining the initial codes, and classifying similar initial codes in more comprehensive categories. ^20^ Four criteria of Lincoln and Guba including credibility, dependability, conformability, and transferability were applied to assess the rigor and trustworthiness of the qualitative data. ^21^

The credibility was investigated through prolonged engagement in data, triangulation of data from interviews and focus group, member checking, and peer debriefing. Two experts in qualitative studies reviewed the interviews, codes, and the extracted categories. To assure the dependability, we explained all the phases and methodology used extensively and two faculty members that were familiar with qualitative research extracted all the codes and the categories. To establish confirm ability, we prepared a detailed report of the process and procedures used in this research to be evaluated by two external evaluators. As to verification of the transferability, sampling was done with the maximum variation approach (in terms of age, place of work, etc.); a precise description of the study context and a rich report of the data were provided to obtain a wide spectrum of outlooks and experiences to make the results as transferable as possible. All members of the research team were involved in data analysis. 

### Second Stage: Two-round Consensus-building Approach

#### Round 1: ‘rate’ the relative importance of the items

Based on individual interviews and a focus group, an online questionnaire was produced. The questionnaire was distributed and collected in Google forms. 

Generally, there is no consensus regarding the panel size needed for a Delphi. ^17^ The qualities of the panelist are more important than the number of panelists, and some research has suggested that about 30 participants are suitable. ^18^ For the current research, because the diversity of opinions was deemed appropriate, there was an attempt to enroll 30 participants in each group to enable feasible number of participants and an equal representation of these groups. 

Based on different economic and socio-cultural regions of Tehran, the panelists were selected using a stratified cluster random sampling method. Tehran was divided into five arbitrary geographic regions: south, north, east, west, and center. Within each area, one male high school was randomly selected from the list of all high schools. In each school, six students were selected based on their educational status; each student with his parents was considered a cluster. In each school, six teachers were selected based on work experience as well.

To determine the importance of each problem, we listed 63 behavioral problems in a questionnaire and presented them to different groups, including 30 students of different grades, 30 teachers, and 30 parents. They were asked to give a grade of 1 to 10 to each problem according to their viewpoint, where 1 meant the least important and 10 the most important. 

After collecting responses from the participants, data were analyzed using SPSS software. The mean of the grades of each problem phrase was calculated for three groups of participants and the problems were sorted accordingly. Thus, the top 20 behavioral problems were identified according to each group’s opinion. 

#### Round 2: ‘Rank’ 

After the estimation of the mean grades of top 20 problem given by all participants in the previous round, respondents were asked to ‘rank’ the ten top problems. 

Agreement of participants is used to determine consensus in a Delphi process, but there is a wide range of definitions for consensus in the literature, and there is no definitive method for the desired level of consensus. ^18^ The definition of consensus chosen for the current study was a minimum of 75% agree or strongly agree with an item.

### Ethical Considerations

The Ethics Committee of Endocrine and Metabolism Research Institute affiliated to Tehran University of Medical Sciences approved the study (IR.TUMS.EMRI.REC.1397.027). The participants were assured that all personal information, opinions, and interviews would be kept confidential and they could withdraw from the study anytime. 

## RESULTS

The participant in the first stage were 24 and the participating panel in the second stage was composed of 90 members. Demographic characteristics of these participants are presented in Tables 1 and 2. 

**Table 1:** Demographic characteristics of the participantsin the first stage

Group	Number	Age	Sex	Education
Teachers	1	28	Male	BSc
	2	39	Male	PhD
	3	45	Male	BSc
	4	42	Male	MSc
	5	36	Male	BSc
	6	50	Male	BSc
	7	29	Male	MSc
Parents	8	39	Male	BSc
	9	39	Female	BSc
	10	45	Female	BSc
	11	53	Male	Diploma
	12	37	Female	MSc
	13	50	Male	BSc
	14	38	Male	PhD
Students	15	17	Male	High school
	16	18	Male	High school
	17	17	Male	High school
	18	17	Male	High school
	19	18	Male	High school
	20	18	Male	High school
	21	18	Male	High school
	22	17	Male	High school
	23	17	Male	High school
	24	18	Male	High school

**Table 2:** Demographic characteristics of the respondents in the second stage

Group	Age Mean±SD	Sex N(%)	Education N(%)	Job experience N(%)
Teachers	35±4.2	30(100)Male	BSc: 21(70)	<5 year: 6(20)
			MSc: 8(26.7)	5-10 years:15(50)
			PhD: 1(3.3)	>10 years: 9(30)
				**Number of children N (%)**
Parents	41±6.3	30(50)Male	Diploma: 30(50)	1: 15(50)
		30(50) Female	BSc: 15(25)	2-3: 12(40)
			MSc: 10(16.7)	>3: 3 (10)
			PhD: 5(8.3)	
Students	17±1.1	30(100) Male	30(100%) High school	

### First Stage: Primary Identification of Problems

The results of data analysis were grouped into eight main categories and 19 subcategories (Table 3).

**Table 3:** Main categories and subcategories from content analysis of text data about behavioral concerns

Categories	Subcategories	Categories	Subcategories
Relational challenges with parents and other adults	Emotional distance from parents	Self-esteem and sense of purposefulness issues	Lack of the purpose and motivation
	Lack of effective communication with other adults		Lack of self-confidence
Poor interaction with peers	Odd friendships	Materialistic tendencies	Luxurious lifestyle
	Inappropriate interactions with peer groups		Economic needs
	Opposite sex friendships		Fashion orientation
Lack of adaptation to conditions	Complaining	Cyberspace issues	Wasting time at cyberspace
	Not paying attention to one’s situation		Unsafe presence in cyberspace
	The lack of problem-solving skills		Behavioral anomalies
Emotional dysregulation	Low threshold concerning the negative emotions	Non-adherence to religious belief or practice	Lack of knowledge about religious principles
	Superficial conflicts		Lack of commitment to religious and spiritual beliefs

### 1. Relational Challenges with Parents and Other Adults

This category, as one of the most important categories of the qualitative stage, has two subcategories. 

#### 1.1. Emotional Distance from Parents

As children reach adolescence, the emotional distance between them and their parents seems to be increasing. Adolescents expect their parents to listen to them, and support and help them. One of the parents said:

*“A healthy and trustful relationship between parents and their children is of the greatest importance during adolescence. Of course, having a close relationship with teenagers is not an easy task. If teenagers feel that their parents interfere with their own affairs, they will not have good reaction, but they talk to their friends constantly via text messages and social media…. On the other hand, when their mother asks questions, they may not respond.”* (P. 11)

One of the teachers said: *“At this age, teens make decisions that have major consequences on their life trajectory, such as choosing a friend, behaving differently at school or when driving a car, smoking cigarettes and opposite sex friendships…. The parents’ constant criticisms threaten the core of adolescents’ personalities. These teens gradually increase the distance between themselves and their parents emotionally.”* (P.3)

One of the points repeatedly reported by adolescents is their parents’ lack of understanding, which can lead to the emotional distance between them and their parents. One of them said, *“Unfortunately, my parents do not probably remember how to behave when they were a teenager. They repeatedly give advice and tell me why I wear these clothes… or why I go out with my friends. Therefore, when such arguments occur among us, I try not to talk to them for a few days.”* (P.15)

#### 1.2. Lack of Effective Communication with Other Adults

In modern society, changes in lifestyles have completely ignored many values, which were important in the past. One of these values is respect for other adults. 

One of the parents said, 

*“In the past, teenagers were much better than today’s teens. Time has changed and if you faint on the bus, no teenager will offer you a seat.”* (P.13)

### 2. Poor Interaction with Peers

According to Erikson’s theory, one of the most important success factors for the adolescents is the attachment to peers and belonging to the peer groups. For this reason, one of the categories was poor peer interactions and friendships, which consisted of three important subcategories.

#### 2.1. Odd Friendships

Families played a dominant role in the past two decades, and adolescents had more interactions with their parents. However, today, adolescents develop their relationships with the peer groups. One of the problems pointed out by parents and teachers is the lack of proper knowledge of the world around them and adolescents’ interactions with the peer groups.

A teacher said, 

*“Sometimes, the nature of this type of relationships is a healthy friendship, but teenagers suffering from the emotional gap between them and one or both parents may engage in odd friendships and in some cases…, its negative impact is even greater than that of the emotional distance. They become extremely vulnerable and are forced to get friends with everyone.”* (P.2)

#### 2.2. Inappropriate Interactions with Peer Groups

Mockery is an ugly habit that is commonly seen among some people, especially teenagers. During adolescence, mocking each other is known as a typical pastime of the teenagers, especially teenage boys. Obviously, derision is more than just making fun of someone, which can destroy relationships with friends. When a person is ridiculed, he/she stays away from the person who ridicules him/her. His/her friends gradually desert a person who constantly mocks and derides them and he/she becomes isolated and lonely. Another teacher said:

*“No one likes to be associated with someone who constantly mocks and derides others. Teenagers avoid drawing attention to themselves in public for fear of being ridiculed, and this is one of the causes of social isolation and loneliness in adolescence.”* (P.7)

#### 2.3. Opposite Sex Friendships 

Adolescence begins with puberty. Individuals become more interested in forming opposite-sex relationships that can determine their fate. The type of adolescents’ relationships with the opposite sex during puberty and its effect on their physical, intellectual, and emotional growth will have an undeniable effect on choosing their future spouse. 

Another parent said, 

*“The opposite sex instinctis natural and normal for the adolescents. As adolescents reach puberty, the majority of them fall in love at first sight and think it is a real love, while it is not as meaningful and enduring as love is over time. Many teenagers indulge the romantic relationships and can get HIV due to the lack of awareness. Sometimes, breakup in the romantic relationship can have serious consequences for teenagers who feel depressed.”* (P. 10)

### 3. Lack of Adaptation to Conditions 

Rebelling in teenagers is one of the characteristics of adolescence that occurs due to internal and external contradictions, leading to feelings of nervousness. Adolescents have always tried to adjust their environment to their desires, which in case of failure will cause behaviors such as not paying attention to their status, indifference, failure to solve the problem, etc. This category includes two subcategories.

#### 3.1. Not Paying Attention to One’s Situation 

A teenager said,

*“I was born in a 3rd world country and a poor family, and I should not compare my life to the lives of my peers in other countries. When Internet access is provided at home, many people use itand this can create a fantasy world with their ideal conditions, which destroy them.”* (P.19)

#### 3.2. The Lack of Problem-solving Skills

Another teenager said,

*“Rolling stone destroying my life, the problem is always there and always will be. Because it is the only way for human evolution,.. it completes you when you have solved it, so instead of grieving, you should seek to solve it and do not think that if I am busy with other issues, the problem has nothing to do with me… people cannot walk in shoes that are full of thorns.”* (P.20)

### 4. Emotional Dysregulation

This category includes two subcategories. 

#### 4.1. Low Threshold Concerning the Negative Emotions

Their relatively low threshold concerning anger and the occasional conflicts of adolescents are the major concerns of their parents and teachers. One of the parents said, 

*“Adolescence is also a challenging period for parents because adolescents have not yet reached a stage where they can manage their negative emotions and are prone to making bad decisions which have serious consequences. A request that seemed reasonable to dad may be received as a grievous outrage.”* (P.14)

#### 4.2. Superficial Conflicts

Another teacher said, 

*“Teenagers’ conflicts have no benefits sought by them and are superficial. That is, the teenager starts the conflict when it is not serious. For example, when someone looks at them sideways, they conflict with him.”* (P.1)

### 5. Self-esteem and Sense of Purposefulness Issues

According to Erickson’s theory, adolescence is one of the most important stages of cognitive or intellectual development and identity stability; therefore, identity crisis occurs during adolescence. The self-knowledge, self-confidence, absence of despair, responsibility, social interactions, adaptability, creativity, and purposefulness are among the characteristics of an independent identity, according to many experts. During adolescence, sometimes they face challenges due to economic, social, and cultural problems. This category is one of the most important categories of the qualitative stage findings, which includes two subcategories.

#### 5.1. Lack of Purpose and Motivation

A teacher said, 

*“Unfortunately, due to all kinds of social problems such as inflation, unemployment, lack of self-awareness and inappropriate education, most adolescents become emotionally numb and are unmotivated.”* (P.3)

Another parent said, 

*“The loss of motivation among adolescents is one of the most difficult situations that we have faced. If they lack purpose and motivation, it will have major consequences on their life trajectory.”* (P.13)

#### 5.2. Lack of Self-confidence

A teacher said,

*“Adolescents do not have high self-confidence. They do not act independently and rely on another person. They underestimate themselves and their abilities or achievements. They are easily influenced by other people and avoid new situations.”* (P.5)

### 6. Materialistic Tendencies

This category includes three sub-categories. 

#### 6.1. Luxurious Lifestyle

Modern lifestyle formed based on consumerism has affected all aspects of individual and collective behavior, and, in general, all sectors and elements of culture have been affected. This process of change in lifestyle, which some called consumerism or luxurious lifestyle, has a huge influence on the teens. 

One parent said, *“Today, short or long hairstyles, hair styling, etc…. have become a major part of the boys’ lives. In the past, they shaved their heads, but these days, boys lengthen and shorten their hair based on the latest fashions at great prices. However, they consider an increase in their living cost as part of the adolescent stage”*(P.11)

#### 6.2. Economic Needs

Another teenager said, 

*“Well, at this age, we like to go to coffee shops and restaurants with our friends; sometimes, we plan to go to the game nets, and we spend our money on these things. I think our parents don’t pay attention to our pocket money.”* (P.20)

#### 6.3. Fashion Orientation

The adolescence phase is marked by paying attention to fashion, clothing and make-up. In addition, teenagers are interested in wearing clothing styles that are popular among their peers. However, regarding the fashionable trends followed by teenagers, a teenager said, 

*“I do not like to be mocked by others for my appearance. I had to stay alone at classroom because of my dressing style... I could not talk to anyone, which makes me feel bad. Thus, I decided to wear the clothes like today. I cut my hair based on hairstyles and with these changes… I become attracted to my peers and my relations with my friends became stronger and distract myself from the loneliness.”* (P.19)

### 7. Cyberspace Issues

If we look at the number of users in cyberspace, we find that a high percentage of them is teenagers. Cyberspace allows teenagers to receive special attention. If getting a showoff in cyberspace is not properly managed, it can lead to behavioral anomalies. This was another important category, which included three important subcategories.

#### 7.1. Wasting Time at Cyberspace

Apparent said, 

*“The main problem with cyberspace, which is very harmful, is that teen’s surf aimlessly in cyberspace. Time is almost meaningless in cyberspace… This *“timelessness”* disrupts their way of life. They sacrifice their sleep and rest time to being online in cyberspace. They become fundamentally dependent on cyberspace for their way of life. They are unable to concentrate on schoolwork which can lead to academic failure.”* (P.12)

#### 7.2. Unsafe Presence in Cyberspace

Secure and reliable cyberspace is one of the most important sub-categories of the issues related to adolescence, which has received more attention from parents and teachers. 

A teenager said,

*“When we talk to people in cyberspace, they can introduce themselves to us other than what they really are. Thus, we can’t trust these people who may have multiple identities.”* (P.17)

#### 7.3. Behavioral Anomaly

One of the teachers said, 

*“Teens, who spend 24 hours a day playing computer games, chatting, browsing the sites and working with various applications and competing with others in cyberspace,…are no longer interested in interacting with others in the real space. In fact, surfing the cyberspace makes their minds one-dimensional, leading to social skill problems in them. They cannot express their feelings and have cold feelings toward other people; this inability to communicate with different people leads to depression, anxiety and low self-esteem.”* (P.2)

### 8. Non-adherence to Religious Belief or Practice

This category constitutes two sub-categories.

#### 8.1. Lack of Knowledge about Religious Principles

Participants believed that they are less aware of the shari’aprinciples regarding issues related to puberty and are confused, skeptical, and misguided.

A teenager said,

”*I did not know it had its own rules… when I had a wet dream.”* (P.18)

#### 8.2. Lack of Commitment to Religious and Spiritual Beliefs

A teenager said,

*“Children who are entering adolescence are going through many changes and they can be stimulated by anything. Those who pray show no tendency for anti-religious contents presented in social media. Unfortunately, most of teenagers do not adhere to religious proscriptions against unhealthy behaviors.”* (P.16)

### Second Stage: Two-round Consensus-building Approach

#### Round 1: ‘Rate’ the Relative Importance of the Items

63 identified behavioral problems for round 1 are presented in Table 4. According to the specified scores given to different issues, twenty top problems were extracted (Table 5). 

**Table 4:** Identified behavioral concerns for the second stage (two-round consensus-building approach)

Behavioral concerns categories	Behavioral concerns phrase	Behavioral concerns categories	Behavioral concerns phrase
Relational challenges with parents andother adults	Cold relation with parents	Self-esteem and sense of purposefulness issues	Lack of personality development
	Not to accept the parents’ opinions		Insufficient self-esteem
	Parents’ lack of communicational skills		Hopelessness about future
	Feeling not being understood (parents, teachers)		Feeling incapable of doing a big job
	No respect for adults		Lack of concentration on specific job
	Not respecting the rights of others		Lack of motivation
	Being rejected by adults		Inattentiveness about the society and the country
	Adolescents’ lack of communicational skills		Lack of patriotism
	Lack of knowledge about social norms		Lack of purposefulness
	Lack of verbal interaction and requesting from adults		Lack of planning and prioritization skill
Poor Interaction with peers	Unsafe friendship with abnormal sexual tendencies		Aimlessness
	Making extravagant friendshipwith extreme emotional dependencies		Not having lifelong visions
	Lack of ability to keep privacy		Lack of ideal role model
	Lack of knowledge about respecting friends		Inaction and laziness
	Maintaining human dignity in friendship		Doing things at the last minute
	Mocking each other		Lack of time management
Lack of adaptation to conditions	Complaining	Materialistic tendencies	Wasting
	High expectation		Much emphasize fashion and brands
	Self-egoism		Extreme concern for money
	Lack of adaptation with current situation		Extreme hedonism
	Incompatibility in social life		Not having a proper model of leisure
	Only working in a self-desirable environment		Costly life
	Projecting failures		Lack of insight towards living expenses
	Lack of resilience		Paying much attention to food and clothes
Emotional dysregulation	Extreme use of foul language,	Cyberspace issues	Wasting time at cyberspace
	Extreme anger		Lack of knowledge about safe and secure cyberspace
	Being extremely nervous		Lack of media literacy
	Lack of skill to control stress	Non-adherence to religious belief or practice	Not respecting religious educations
	Lack of knowledge to control anger		No obligation to religious rituals
	Insufficient skill for controlling crisis		No obligation to moral and ethical values
	Sever distress due to a bad score		
	Lack of emotions recognition		
	Lack of knowledge and skills to respect others’ feelings		

**Table 5:** The first twentybehavioral concerns according to different groups’ point of view

Number	Students	Mean Score	Teachers	Mean Score	Parents	Mean Score
1	Lack of planning and prioritization skill	8.67	Costly life	8.00	Wasting time at cyberspace	7.88
2	Wasting time at cyberspace	8.65	Not having a proper model of leisure	7.90	Lack of resilience	7.80
3	High expectation	8.58	Insufficient skill for controlling crisis	7.95	Lack of knowledge about safe and secure cyberspace	7.75
4	Extreme use of foul language	8.42	Much emphasize on fashion and brands	7.88	Lack of knowledge about social norms	7.63
5	Insufficient skill for controlling crisis	8.33	Feeling not being understood (parents, teachers)	7.75	Lack of insight towards living expenses	7.60
6	Lack of time management	8.30	Extreme concern for money	7.70	Doing things at the last minute	7.59
7	Only working in a self-desirable environment	8.29	Lack of concentration on specific job	7.68	Costly life	7.50
8	Lack of motivation	8.25	Extreme hedonism	7.65	Lack of skill to control stress	7.38
9	Inaction and laziness	8.17	Extreme concern for money	7.64	Cold relation with parents	7.20
10	Lack of purposefulness	8.15	Doing things at the last minute	7.63	Not having a proper model of leisure	7.25
11	Mocking each other	8.08	Inaction and laziness	7.62	Not to accept the parents’ opinions	7.30
12	Feeling incapable of doing a big job	8.00	Lack of insight towards living expenses	7.60	Lack of knowledge to control anger	7.28
13	Insufficient self-esteem	7.99	Lack of time management	7.59	Complaining	7.13
14	Extreme hedonism	7.98	Lack of motivation	7.58	High expectation	7.00
15	Projecting failures	7.92	Lack of knowledge about safe and secure cyberspace	7.50	Lack of adaptation with current situation	6.99
16	Lack of knowledge and skills to respect others’ feelings	7.75	Projecting failures	7.49	Not respecting the rights of others	6.90
17	Not having lifelong visions	7.70	Lack of media literacy	7.25	Being extremely nervous	6.80
18	Complaining	7.67	Lack of resilience	7.14	Only working in a self-desirable environment	6.75
19	Wasting	7.50	Lack of skill to control stress	7.13	Parents’ lack of communicational skills	6.70
20	Lack of adaptation with current situation	7.35	Lack of knowledge to control anger	7.12	Extreme concern for money	6.69

#### Round 2: ‘Rank’

At last, ten top problems according to the consensus of different groups are extracted andpresented in Table 6.

**Table 6:** Top ten prioritiesof behavioral concerns according to different groups’ point of view

Priority	Students	Consensus	Teachers	Consensus	Patents	Consensus
1	Lack of planning and prioritization skill	95%	Insufficient skill for controlling crisis	98%	Lack of resilience	97%
2	Wasting time at cyberspace	94%	Not having a proper model of leisure	97%	Wasting time at cyberspace	96%
3	High expectation	91%	Costly life	92%	Lack of knowledge about safe and secure cyberspace	95%
4	Extreme use of foul language	90%	Much emphasize fashion and brands	89%	Lack of insight towards living expenses	94%
5	Lack of time management	88%	Extreme hedonism	84%	Doing things at the last minute	90%
6	Insufficient skill for controlling crisis	82%	Feeling not being understood (parents, teachers)	80%	Lack of knowledge about social norms	89%
7	Only working in a self-desirable environment	80%	Extreme concern for money	78%	Costly life	88%
8	Lack of motivation	78%	Lack of concentration on specific job	77%	Lack of skill to control stress	84%
9	Lack of purposefulness	75%	Lack of time management	76%	Not to accept the parents’ opinions	82%
10	Inaction and laziness	74%	Lack of motivation	75%	Not having a proper model of leisure	75%

## DISCUSSION

This study examined how adolescents, their parents and teachers, prioritized their behavioral concerns. The first aim of the study was to determine the most important priority from the perspective of adolescents. The results of this study highlight the necessity of goal-setting as the most important priority for male adolescents. In addition, controlling behavioral problems and creating the necessary motivation are among the most critical issues indicated as influential by the adolescents. The most 10 important mentioned problems (Table 6) by teenagers could be solved through paying attention to and improving their life skills. Life skills have been identified as an essential resource for developing cognitive, behavioral, emotional, psychosocial, and resilience skills to deal with everyday challenges and constructive involvement in the community. These skills are known to be key elements to dealing and mediating challenges that adolescents face in becoming productive individuals. ^22 , 23^ The results of the studies in Iran have confirmed that most Iranian students lack the necessary life skills, ^24^ and few students have desirable life skills. ^25^ On the other hand, Iran’s Ministry of Education was not successful in teaching life skills in schools. ^12^ Studies reported that the majority of life skills programs in developing countries failed in systematic implementation, evaluation and monitoring. Courses are often conducted to yield short term outcomes only. ^26^

Generally, these investigations have given special value to motives such as revisions of the nature and definition of life skills, redrafting the theoretical bases of educational programs and their governing approaches, careful attention to gender differences, evaluation of practical competence of teachers, administrative compliance obligation, and creation of an age-stratified conceptual design for elementary to high school students. 

Since several studies have highlighted the role of teaching life skills in decreasing deviant behaviors and increasing positive and constructive behaviors, ^12 , 25^ revising the old definitions and using expert teachers for teaching life skill programs could obviously decrease the behavioral problems of adolescents, especially male ones. Moreover, adolescents may benefit from programs that target emotional competencies. Emotional competencies include knowledge and skills necessary to set and achieve the goals, understand and manage emotions, show compassion and empathy for others, and establish and keep positive relationships. ^27^

The second aim of the study was to compare the most important priority from the perspective of adolescents, parents, and teachers. In this vein, ten top general concerns have approximately a similar structure among groups; however, each group member marks the first priority quite differently. Accordingly, “lack of planning and prioritization skill” was the most important priority for male adolescents, while the majority of parents prioritized “lack of resilience” as the most important problem. In contrast, teachers marked the “insufficient skill for controlling crisis” as the most important problem identified, which could be attributed to the differences in attitudes and different needs, which each group has. 

There are a number of challenges, such as drugs and substances abuse, violence, emotional traumas, sexual experience, and other problems that make the adolescence period especially dangerous. This transitional period is especially important for the development of healthy adaptation competencies. The growth in personal adaptation abilities of an adolescent to maintain or regain positive outcomes despite stress or negative emotional experiences is seriously influenced by defense factors (resiliency factors). ^28^ No studies have been published measuring Iranian adolescents’ resiliency, because of few specific questionnaire focusing on resilience in adolescents; ^29^ however, the role of family as a source of constant support of adolescent’s resiliency is well known, ^28^ which was also confirmed by parents in this study.

Other important priorities reported by adolescents and their parents were “wasting time at cyberspace” and “lack of knowledge about safe and secure cyberspace”. In a study conducted with the aim of describing the psychosocial experiences of Iranian adolescents in the Internet’s virtual space, the adolescents’ negative and positive experiences formed based on personal and environmental factors. Perceived threats, decline of behavioral values and principles, temptation, emotional and social helplessness, increase in the social capital, escape from loneliness, a good feeling in life, visibility in the social network, and intelligent selection of content were formed. ^30^ However, in problematic Internet use, there is a need for promoting preventive interventions in the school setting. ^31^

In the meantime, the result of data collection on parents and teachers’ groups revealed a similar pattern in terms of the identified problems. By looking at these findings, it is inferred that the priorities of male adolescents are more internal issues, and those of the parents and teachers are more external, social, and communication issues. Research shows that the health literature often considers men as emotionally repressed and boys are forced to be silent and tough. Moreover, the rates of help seeking, especially in older adolescent boys aged 16–20 years, are low. ^32^

To sum up, it can be concluded from the results of this study that four aspects of improving self-esteem, empowering for safe presence at cyberspace, enhancing personality management skills and purposefulness, and improving emotion control are the most important aspects, which can be focused by experts (teachers, families ...) while designing interventional programs. 

In similar studies, other social and political issues such as focusing on support policies, providing counseling services, paying attention to justice and equality in meeting the adolescents’ needs, supporting national and international interventions, generalizing financial supports in providing health services for the adolescents, using prevention strategic policies, and controlling the adolescents’ issues are mentioned as the predominant ones. ^16^

Therefore, while dealing with adolescents, it is necessary to use practical guidelines. The next step will be designing practical programs according to their educational performance and determining intervention structures based on the existing situation. After implementing practical programs, an important job will be concentrating on evaluation measures and assessing the effectiveness of interventions for attaining the objectives. 

In addition to the important implications raised by the present study for the consideration of policy makers, these finding scan also be used as a good conceptual understanding for identifying and classifying the adolescents’ behavioral concerns in similar cultures. Certainly, regional and local stakeholders and policy-makers can utilize these findings for designing effective interventions at school or regional level.

This study, like most qualitative studies, had some limitations; the results are not generalizable to all adolescents, parents, and teachers. This design was selected to obtain different opinions of adolescents, parents and teachers and they are not representative of all participants’ vision beyond all schools. Nevertheless, increasing the sample size and extending the interviews to include other participants from various cultures would be of great interest. Despite the limitations, one of important strong points of the present study is that it takes into consideration three views on behavioral concerns, including those of adolescent, family and school. Future research should explore how actions would be helpful about behavioral concerns of the adolescent people. 

## CONCLUSION

We engaged teachers, adolescents, and their parents in the process of prioritization of behavioral problems. The finding of this study showed “lack of planning and prioritization skill”, “insufficient skill for controlling crises”, and “lack of resilience” as the most important priority for male adolescents, teachers, and parents, respectively. Decision-makers and policy- makers can consider the findings of this study.
